# Regulation of Tolerogenic Features on Dexamethasone-Modulated MPLA-Activated Dendritic Cells by MYC

**DOI:** 10.3389/fimmu.2019.01171

**Published:** 2019-05-28

**Authors:** Paulina A. García-González, Jaxaira Maggi, Katina Schinnerling, Alejandro Sepúlveda-Gutiérrez, Lilian Soto, Oscar Neira, Ahmed M. Mehdi, Hendrik J. Nel, Bárbara Pesce, Octavio Aravena, María Carmen Molina, Diego Catalán, Ranjeny Thomas, Ricardo A. Verdugo, Juan Carlos Aguillón

**Affiliations:** ^1^Programa Disciplinario de Inmunología, Facultad de Medicina, Instituto de Ciencias Biomédicas, Universidad de Chile, Santiago, Chile; ^2^Programa de Genética Humana, ICBM, Facultad de Medicina, Universidad de Chile, Santiago, Chile; ^3^Unidad de Dolor, Departamento de Medicina, Hospital Clínico Universidad de Chile, Santiago, Chile; ^4^Sección de Reumatología, Hospital del Salvador, Santiago, Chile; ^5^Diamantina Institute, Translational Research Institute, Princess Alexandra Hospital, University of Queensland, Brisbane, QLD, Australia; ^6^MED.UCHILE-FACS Laboratorio, Instituto de Ciencias Biomédicas, Facultad de Medicina, Universidad de Chile, Santiago, Chile

**Keywords:** tolerogenic dendritic cells (tDC), tolerance mechanism, DC transcription factors, zinc metabollism in DC, dexamethasone-modulated and MPLA-activated DC, ROS metabollism in DC

## Abstract

The potential of tolerogenic dendritic cells (tolDCs) to shape immune responses and restore tolerance has turn them into a promising therapeutic tool for cellular therapies directed toward immune regulation in autoimmunity. Although the cellular mechanisms by which these cells can exert their regulatory function are well-known, the mechanisms driving their differentiation and function are still poorly known, and the variety of stimuli and protocols applied to differentiate DCs toward a tolerogenic phenotype makes it even more complex to underpin the molecular features involved in their function. Through transcriptional profiling analysis of monocyte-derived tolDCs modulated with dexamethasone (Dex) and activated with monophosphoryl lipid A (MPLA), known as DM-DCs, we were able to identify MYC as one of the transcriptional regulators of several genes differentially expressed on DM-DCs compared to MPLA-matured DCs (M-DCs) and untreated/immature DCs (DCs) as revealed by Ingenuity Pathway Analysis (IPA) upstream regulators evaluation. Additionally, MYC was also amidst the most upregulated genes in DM-DCs, finding that was confirmed at a transcriptional as well as at a protein level. Blockade of transactivation of MYC target genes led to the downregulation of tolerance-related markers IDO1 and JAG1. MYC blockade also led to downregulation of PLZF and STAT3, transcription factors associated with immune regulation and inhibition of DC maturation, further supporting a role of MYC as an upstream regulator contributing to the regulatory phenotype of DM-DCs. On the other hand, we had previously shown that fatty acid oxidation, oxidative metabolism and zinc homeostasis are amongst the main biological functions represented in DM-DCs, and here we show that DM-DCs exhibit higher intracellular expression of ROS and Zinc compared to mature M-DCs and DCs. Taken together, these findings suggest that the regulatory profile of DM-DCs is partly shaped by the effect of the transcriptional regulation of tolerance-inducing genes by MYC and the modulation of oxidative metabolic processes and signaling mediators such as Zinc and ROS.

## Introduction

The ability of dendritic cells (DCs) to modulate immune responses and educate the immune system to induce tolerance, has led to a wide range of studies of these cells as targets for cellular therapy in autoimmunity and other disorders where immune tolerance is broken. These tolerogenic DCs (tolDCs), are capable of inducing anergy or deletion of effector T cells, as well as the differentiation and/or proliferation of regulatory T cell (Treg) subsets and the establishment of a local anti-inflammatory milieu. Regulation may result from various processes, including deficient antigen presentation, reduced co-stimulatory molecules, expression of inhibitory molecules and/or secretion of anti-inflammatory cytokines such as IL-10 and TGF-β (1, 2). To this date, many *in vitro* differentiation protocols of tolDCs from blood monocytes have been published, which include the use of a wide variety of immunomodulatory stimuli to induce a regulatory profile on DCs (3–7). Although some features may differ between tolDC subsets, all are endowed with the capacity to exert regulatory functions (8, 9). The main idea is to *ex vivo* differentiate precursor cells from peripheral blood of patients to DCs, endow them with regulatory features, load them with a specific antigen, and then administrate them to the patient, in order to restore immune tolerance in an antigen-specific manner.

Keeping this on mind, our group developed a protocol for the generation of tolDCs from peripheral blood monocytes further modulating DCs with dexamethasone (Dex) to induce a tolerogenic phenotype, followed by an alternative activation with the non-toxic LPS analog monophosphoryl lipid A (MPLA), named DM-DCs. These cells display reduced levels of surface markers CD83 and CD86, secrete high amounts of IL-10 and TGFβ, show lymph node homing capacity and exhibit a reduced capacity to promote effector Th1 and Th17 cell proliferation, besides being able to render these cells hypo-responsive in an antigen-specific manner while remaining stable in front of pro-inflammatory stimuli (10, 11).

While the mechanisms by which tolDCs can exert their immunomodulatory actions have been broadly studied, the molecular setup that leads to the differentiation of DCs into a regulatory profile, is much less understood, and the fact that different tolerogenic stimuli can generate different tolDC subsets makes it even harder to identify the molecular components accountable for immune regulation in tolDCs, since different stimuli activate different signaling pathways that can lead to tolDCs differentiation. Recent technological advances in the last few years mostly in the “omics” field, along with the advent of multiparametric flow cytometry combined with bioinformatics analyses, have made it possible to acquire a deeper insight into the molecular characterization of DC biology. Using these techniques, through genome-wide transcriptional analysis complemented by multi-parametric flow cytometry, we demonstrated that DM-DCs exhibited a transcriptional and phenotypic profile that clearly distinguished them from other monocyte-derived DC (moDC) subsets, such as MPLA-matured DC (M-DCs), Dex-modulated DC (D-DCs) and untreated/immature DC (DCs) (2, 12). These cells were further characterized by the upregulation of several tolerance-related molecules such as IDO1 (indoleamine 2,3-dioxygenase 1), IL-10, MERTK (receptor tyrosine kinase), FCGR2B (Fc fragment of IgG, low affinity IIb), C1Q (complement C1q) and JAG1 (Jagged 1); and the downregulation of maturation/inflammation associated markers CD1c, IL-12, FCER1A (Fc fragment of IgG, alpha polypeptide), and DC-SCRIPT (DC-specific transcript protein) (12).

In this work, using the same experimental approach, we focused on the identification of molecular regulators of DM-DCs profile as well as the main biological functions represented on these cells, which might lead to the regulatory phenotype of DM-DCs. We further identify MYC as a key regulator of tolerance-related genes in DM-DCs, and ROS production and zinc homeostasis as main metabolic processes activated on these tolDCs.

## Materials and Methods

### Blood Samples

Fifteen buffy coat samples from healthy subjects were obtained from the Clinical Hospital of the University of Chile. All subjects signed an informed written consent and all procedures were approved by the Ethics Committees for Research in Human Beings from the Faculty of Medicine and from the Clinical Hospital of the University of Chile. Demographic characterization of healthy controls is detailed in Table S1.

### Generation of Monocyte-Derived Dendritic Cell Subsets

Human moDCs were generated from monocytes as previously described (10). Monocytes were isolated from peripheral blood by negative selection using RosetteSep Human Monocytes enrichment cocktail (Stemcell Technologies, Vancouver, Canada) according to manufacturer's instructions. Monocytes were cultured at 2 × 10^6^ cells/ml in serum-free AIM-V medium (Gibco BLR, Grand Island, NY, USA), supplemented with 500 U/ml of recombinant human GM-CSF and IL-4 (eBioscience, San Diego, CA, USA) for 5 days at 37°C and 5% CO_2_. At day 3, culture medium was replenished and cells were incubated with dexamethasone (Sigma-Aldrich, St. Louis, CO, USA) at a final concentration of 1 μM. At day 4, cells were stimulated with 1 μg/ml of cGMP-grade MPLA (Avanti Polar Lipids Inc., Alabaster, AL, USA) (DM-DCs). Unstimulated cells (DCs) and MPLA-matured DCs (M-DCs) generated in the absence of dexamethasone were used as controls of immature and mature DCs, respectively. On day 5, cells were harvested and characterized by flow cytometry. Monocyte purity and gating strategy for DC characterization are shown on Supplementary Figure S1.

### Flow Cytometry

Antibodies used for analysis were anti-human CD11c BUV395 (clone B-ly6), CD83 BUV737 (clone HB15e), STAT1 Alexa Flúor 647 (clone 1/Stat1), STAT3 PerCP-Cy5.5 (clone M59-50) (BD Biosciences); IDO1 PECy7 (clone eyedio), TSC22D3/GILZ PE (clone CFMKG15) (eBioscience); CD86 BV650 (IT2.2), CD1c BV510 (clone L161), MERTK BV421 (clone 590H11G1E3), ZBTB16/PLZF PE (clone Mags.21F7), CD3 BV711 (clone SK7), CD4 Alexa Fluor 700 (clone OKT4), CD25 PE (clone M-A251), IFNγ PE/Cy7 (clone 4S.B3), and TNFα BV605 (clone Mab11) (BioLegend) and P65/RELA PE (APC) (clone 14G10A21); JAG1 Fluorescein (clone 188331) and MYC PerCP (clone 9E10) (R&D Systems). Prior to antibody staining, cells were labeled with Fixable viability dye FVD eFluor 780 (eBioscience). Cells were resuspended in PBS supplemented with 10% of fetal bovine serum (FBS) (HyClone Thermo Scientific, Logan, UT, USA), stained with specific antibodies, fixed with IC fixation buffer (eBioscience) and resuspended in FACSFlow buffer (Becton Dickinson, San Diego, CA, USA) for subsequent analysis. For intracellular cytokine secretion, cells were treated with 50 ng/ml PMA, 1 ug/ml ionomycin and 1 ul/ml brefeldin A for 5 h. After harvest and surface staining, cells were fixed with IC fixation buffer prior to incubation of antibodies against IFNγ and TNFα in permeabilization buffer (eBioscience). After washing, cells were resuspended in FACSFlow buffer (Becton Dickinson, San Diego, CA, USA) for analysis in the flow cytometer. Data were acquired on a LSR Fortessa X-20 with FACSDiva v6.1.3 software (both Becton Dickinson) and analyzed by FlowJo software (Treestar, USA).

### RNA Isolation

RNA was isolated from 5 × 10^5^ DCs on day 5 using total RNA isolation RNeasy Mini Kit (Qiagen, Hilden, Germany) following the manufacturer's instructions. Yield and quality of RNA samples were evaluated with NanoDrop 1000 spectrophotometer (Thermo Scientific, Waltham, MA, USA) and RNA integrity (RIN score) was analyzed with Agilent 2100 Bioanalyzer (Agilent Technologies, Santa Clara, CA, USA) or LabChip GX/GX II (Caliper LifeSciences, Hopkinton, MA, USA).

### Microarray Analysis

A total of 40 samples, corresponding to 10 healthy donors under 4 experimental conditions, were considered for microarray analysis. Design and data preparation and analysis are detailed in Garcia-Gonzalez et al. (12).

### Confirmation of Gene Expression by qRT-PCR

cDNA was prepared from moDCs RNA samples using the Superscript II Reverse Transcriptase kit (Thermo Fisher Scientific). Quantitative RT-PCR was performed in Stratagene Mx300P, using Brilliant II SYBR Green QPCR Master Mix (Agilent Genomics) with primer sets from IDT. The housekeeping genes Glyceraldehyde 3-phosphate dehydrogenase (*GAPDH*) and 18S ribosomal RNA (*r18S*) were used as internal controls and target gene expression was normalized using untreated DCs expression values. Primer sequences for each target gene are described on Table S2.

### MYC Blockade on DCs

To study the role of MYC on DC phenotype, on day 3 of culture, 3 h after Dex treatment, DCs were incubated with the small MYC inhibitor 10058-F4 (Sigma Aldrich), 30 uM for 48 h until the end of culture. After harvest, cells were washed and stained with antibodies specific for tolerogenic and inflammatory markers analysis through flow cytometry. As control, DCs were also incubated with DMSO.

### Functional Assay

Functional regulatory capacity of DM-DCs was assessed by co-culturing DCs with allogeneic CD4+ T cells, which were isolated from peripheral blood using the RosetteSep CD4+ human T cell enrichment cocktail (Stemcell Technologies, Vancouver, Canada) and labeled with carboxyfluorescein diacetate succinimidyl ester (CFSE) (Sigma-Aldrich) at a concentration of 5 uM. On day 5 after harvest, 10058-F4-treated and untreated DM-DCs were seeded in a 96-well U-bottom plates and co-cultured with CFSE-labeled CD4+ T cells at a 1:2 (DC:T cell) ratio in RPMI medium supplemented with 10% heat inactivated fetal calf serum, at 37°C and 5% CO_2_ for 6 days, after that, CFSE dilution was analyzed by flow cytometry as a measure of proliferation, in addition to IFNγ and TNFα production.

### Determination of Zinc Intracellular Levels in DCs

Zinc influx in DCs was assessed using the cell-permeant zinc fluorescent indicator Newport Green DCF (NG-DCF). Once harvested, cells were washed then fixed and permeabilized prior to staining with 3 uM of Newport Green DCF and an anti-human CD11c antibody. Cells were immediately analyzed at the flow cytometer FACSAria III with FACSDiva v6.1.3 software.

### ROS and Superoxide Detection in DCs

Total ROS and superoxide production in cells was assessed with two fluorescent cell-permeable reagents using the kit Cellular ROS/Superoxide Detection Assay (Abcam). Cells were first stained with CD11c as described above and then fixed. The kit Cellular ROS/Superoxide Detection Assay (Abcam) was then used to stain the cells following the manufacturer's instructions. After staining, cells were immediately analyzed at the flow cytometer FACSAria III with FACSDiva v6.1.3 software.

### Data Exploration and Statistical Analyses

For flow cytometry and qPCR data, Friedman repeated measures test and Dunn's *post hoc* test were used for data comparison between moDC culture conditions. For functional analyses we used a parametric *t*-tests for data comparison. Analyses were performed using Prism 5.01 software (Graphpad, San Diego, USA).

For microarray data analysis differentially expressed genes in modulated DCs relative to unstimulated DCs, were identified with the Maanova package v1.36.0 *t*-test for gene pairwise comparisons (13) and *p*-values were adjusted using false discovery rate method (FDR). Genes with adjusted *p* ≤ 0.05 were considered differentially expressed. Upstream regulators analysis as well as overrepresentation of biological functions was assessed using Ingenuity Pathway Analysis (Ingenuity Systems, Qiagen, Hilden, Germany).

## Results

### Identification of Transcription Factors Governing Gene Expression on DM-DCs

**Figure 1:**
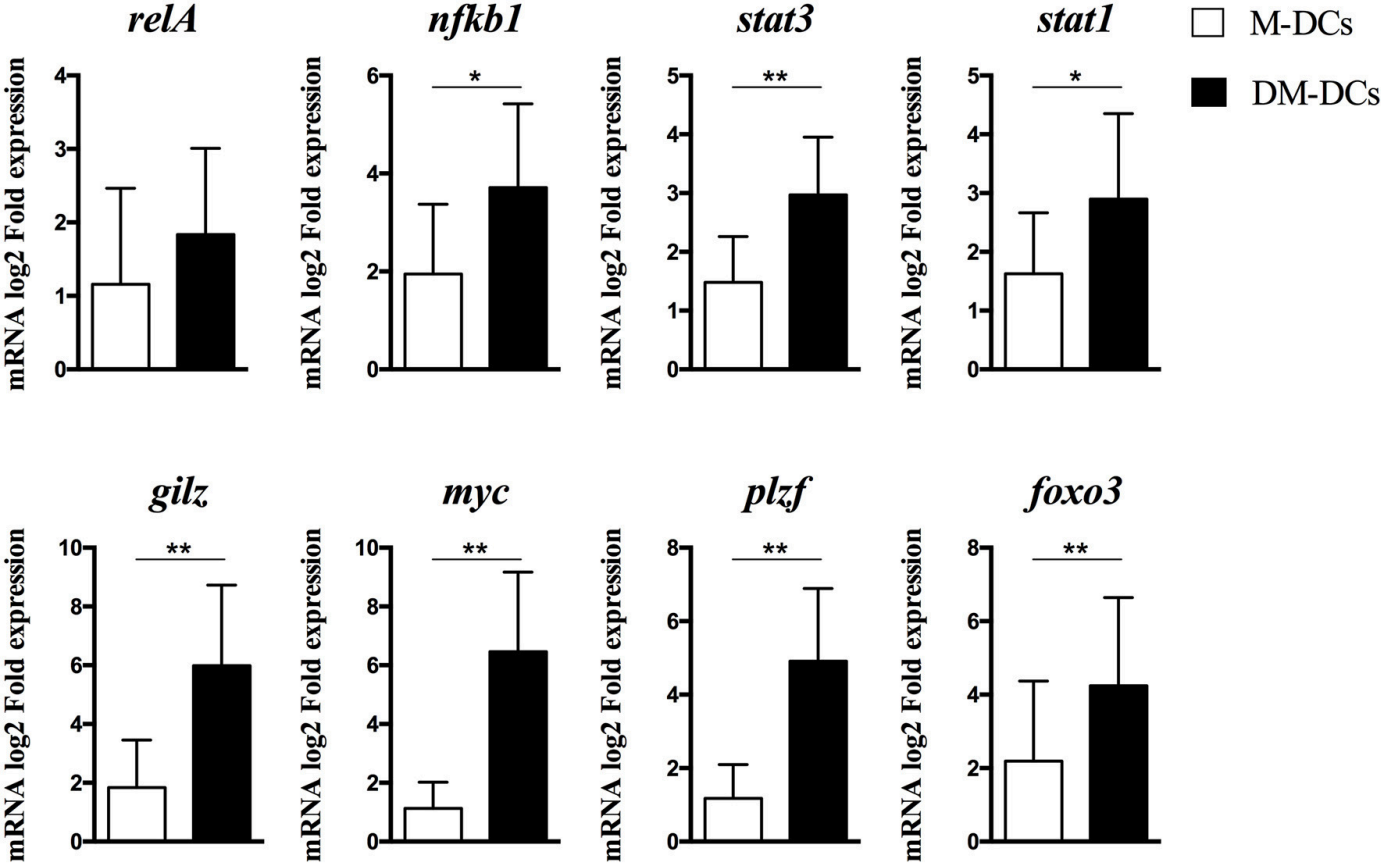
Gene expression levels of main transcription factors described as upstream regulators in Dex-modulated and MPLA-activated dendritic cells (DM-DCs). mRNA levels of transcription factors potentially involved in DM-DCs upstream regulation of gene expression as determined by IPA analysis was assessed by real time PCR. Shown are fold expression values of MPLA-matured DCs (M-DCs) and DM-DCs calculated relative to untreated DCs expression (DCs) (*n* = 10; *p = 0.05, **p = 0.01).

We have previously shown that treatment of human monocyte-derived DCs with dexamethasone and MPLA (DM-DCs) induces a tolerogenic profile, that distinguishes them from other DC subsets at both phenotypic and transcriptional level, and provides them with the potential to exert immune regulatory functions (10, 12). Through genome-wide transcriptional analysis of these cells we found many tolerance-related genes such as IDO1, MERTK, JAG1, FCGR2B, IL-10, and C1Q to be amongst the most upregulated genes in DM-DCs when compared to MPLA-matured DCs and untreated/immature DCs (DCs) (12). Further analysis of differentially expressed genes on DM-DCs using Ingenuity Pathway Analysis allowed us to identify transcription factors acting as upstream regulators in these cells (Table 1). In addition to act as upstream regulators of a large number of genes expressed on DM-DCs, several transcription factors shown on Table 1, are also upregulated on DM-DCs, with fold change values above 1.2, such as gilz (glucocorticoid-induced leucine zipper), myc (avian myelocytomatosis viral oncogene homolog), stat1 (signal transducer and activator of transcription 1), stat3 (signal transducer and activator of transcription 3), plzf (promyelocytic leukemia zinc finger) and nfkb1 (nuclear factor kappa b subunit 1). Real time PCR analysis of these genes confirmed their differential expression on DM-DCs, which presented higher mRNA levels than the mature MPLA-DCs (M-DCs) (Figure 1) and immature/untreated (DCs) controls (data not shown).

**Table 1:** Main transcription factors identified as upstream regulators of differentially expressed genes in DM-DCs.

**Upstream regulator**	**Exp fold change**	***p*-value of overlap**	**Target molecules in dataset**
GILZ	2,332	3,48E-02	CCL5, DUSP1
CEBPD	1,985	1,05E-02	IL1B, MYC
MYC	1,881	6,50E-03	BCL6, GADD45B, ID2, IL17RB, JUN, MYC, SAT1, SHMT1, SPP1, TMSB10/TMSB4X
FOXO3	1,579	4,20E-03	CCND2, CCNG2, CLDN1, CXCL10, EGR2, GADD45B, IER3, JAG1, SGK1, SOD2
STAT1	1,312	8,41E-08	CD14, CD40, CXCL10, CXCL9, IDO1, IFI27, IFI6, IFIT1, IFITM1, IRF7, MX1, MYCSTAT1
TCF7	1,277	6,66E-03	CEBPA, CEBPD
STAT3	1,212	1,26E-02	CD40, CD83, CDH2, CXCL10, CXCL9, EGR2, FSCN1, , JAG1, MUC1, MYC, NAMPT, PTAFR, SGK1, SLAMF1, STAT1
VDR	1,177	4,16E-03	CD14, IFI44L
NFKB1	1,115	1,52E-03	CCL5, CD59, CXCL10, CXCL9, FSCN1, IL1B, IL2RA, IRF4, MYC
SMARCA4	1,079	8,56E-07	CCR6, CD52, DHRS9, ESPNL, FCGR2A, FCGR2B, GPR183, IFIT1, IFITM1, IFITM3, IL1B, JUN, MFGE8, MT1H, MUC1, MYC, TACSTD2, TREM2
CTNNB1	1,132	1,67E-02	ECM1, F13A1, IDO1, IFITM1, MMP7, MYC, SGK1
SMAD4	1,079	1,97E-02	CCL20, CCNG2, FSCN1, GADD45B, IRAK3, JAG1, NAMPT, THBS1
E2F3	1,068	4,04E-06	CCL20, MT1F, MT1G, MT1H, MT1X, MYC, S100A8, THBS1
HIF1A	1,110	1,41E-03	CD24, CLDN1, FSCN1, GADD45B, IRS2, MYC, SDC4, SOD2
EZH2	1,101	3.40E-02	CRISPLD2, CXCL10, GPR68, MYC, RAP1GAP, SLC1A3
STAT5A	1,076	8,26E-04	CD24, CXCL5, GSTT1, MMP7, TACSTD2, TNFRSF6B
NUPR1	1,040	1,60E-02	CXCL5, FOXO3, IRS2, MMP12, MT1X, MX2, MYC, RILPL2, SLC16A10, SLC39A8, XBP1
RELA	1,017	3,87E-04	CCL20, CCL5, CD59, CEBPB, CXCL10, CXCL9, FSCN1, IL1B, IRF4, MYC, NAMPT, SOD2

Except for MYC, almost all the other transcription factors identified have a known association with immune regulation; mainly induction of expression of tolerance-related genes, Treg induction, inhibition of DC maturation or promotion of regulatory responses (14–17). Interestingly, MYC experimental Fold change value was shown to be the highest after GILZ in both microarray analysis and qPCR data (Table 1 and Figure 1).

### Regulation of Immune Regulatory Markers on DM-DCs by MYC

Since IPA analysis revealed that a large number of molecules from the dataset were predicted to be targets of this transcription factor and, since MYC gene expression was also predicted be regulated by several other transcription factors described as main upstream regulators of DM-DCs transcriptome (Table 1), we decided to evaluate its involvement in DM-DCs biology.

**Figure 2:**
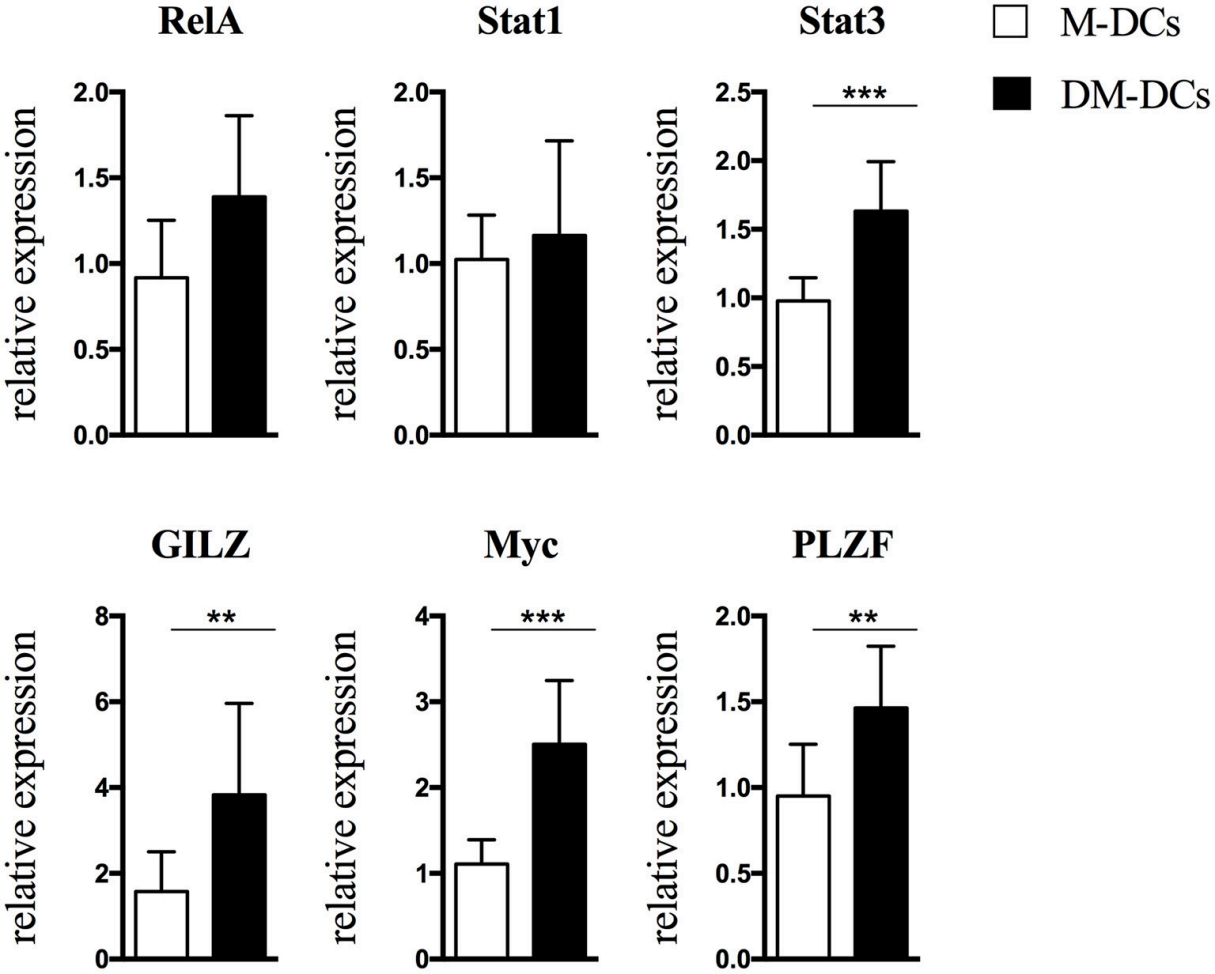
Protein levels of transcription factors involved in regulation of Dex-modulated and MPLA-activated dendritic cells (DM-DCs) gene transcription correlate with gene expression. Protein level of transcription factors potentially involved in upstream regulation of gene expression of DM-DCs was determined by flow cytometry analysis. Shown are relative expression data of MFI values of M-DCs and DM-DCs respect to untreated DCs MFI values (*n* = 10; **p = 0.01; ***p = 0.001).

As depicted in Figure 2 and Supplementary Figures S2, S3, in concordance with its gene expression, MYC protein levels were shown to be higher in DM-DCs than in M-DCs.

**Figure 3:**
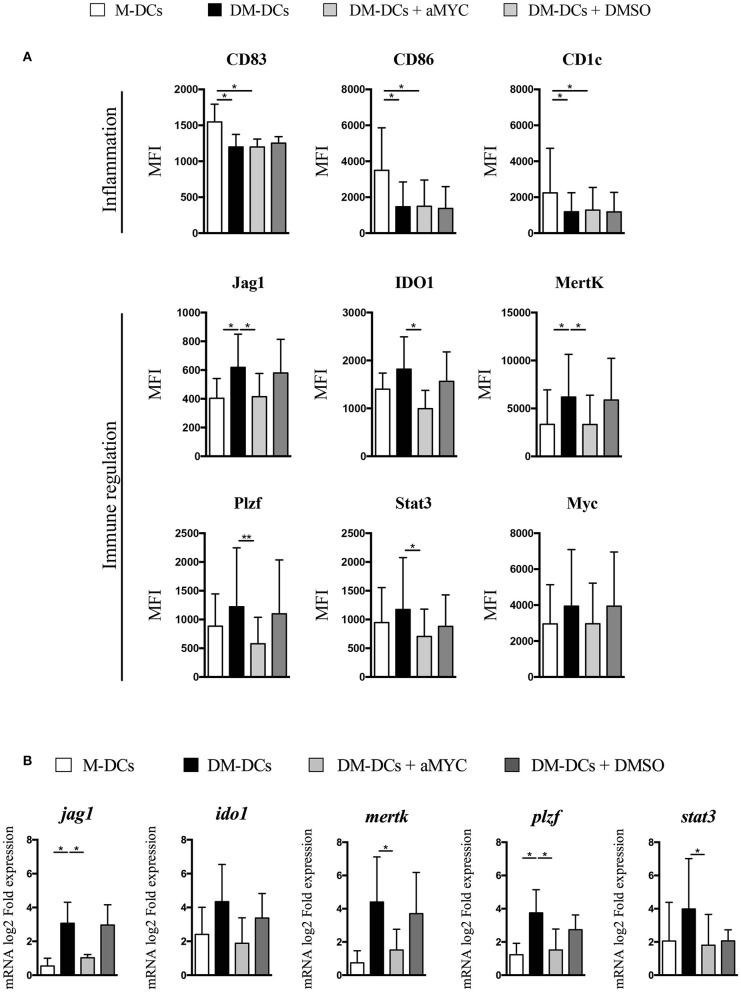
Blockade of MYC leads to inhibition of tolerance-associated gene expression. MYC transactivation of target gene was blocked with the small inhibitor 10058-F4. On day 3 cells were incubated with 10058-F4 (30 μ M) for 48 h, after which immune tolerance markers expression was evaluated at the transcriptional and protein level. **(A)** Flow cytometry analysis of molecules involved in immune response. Shown are MFI values of 5 independent experiments (*n* = 5). **(B)** Gene expression of tolerance-related genes was assessed through real time PCR. Shown are fold expression values of Dex-modulated and MPLA-activated dendritic cells (DM-DCs) with or without 10058-F4 calculated relative to untreated/immature DCs (DCs) expression. M-DCs and DMSO-cultured DM-DCs were used as controls (*n* = 3) (*p = 0.05, **p = 0.01). DM-DCs, Dex-modulated MPLA-activated DCs+; M-DCs, MPLA-matured DCs.

MYC overexpression and association with differentially expressed genes in DM-DCs suggests a potential involvement in DM-DCs regulatory function, so we set to determine if its blockade affected DM-DCs tolerogenic phenotype. For this purpose, we used the small cell-permeable inhibitor 10058-F4, which blocks the transactivation of MYC target genes by specifically blocking the interaction of Myc with its partner Max to form heterodimers which drive gene transcription. Myc blockade for 48 h did not exert any effect on DC maturation/inflammation markers CD83, CD86 and CD1c (Figure 3A and Supplementary Figure S3), indicating that this transcription factor is not involved in the regulation of gene transcription of these molecules. However, we found a robust reduction on protein levels of the tolerance markers JAG1, IDO1 and MERTK, in some cases reaching levels similar to M-DCs (Figure 3A). Gene expression analysis also showed a reduction in mRNA levels of these genes, implying that their expression could be under the control of Myc (Figure 3B). Furthermore, the expression of other transcription factors upregulated on DM-DCs and associated with immune regulation such as PLZF and STAT3, was also affected by the inhibition of Myc (Figure 3B and Supplementary Figure S3). Thus, Myc expression on DM-DCs associates with their tolerogenic phenotype by directly modulating the expression of tolerance markers, and indirectly by regulating the expression of other transcription factors involved in immune regulation.

### Modulation of DM-DCs Regulatory Function by MYC

**Figure 4:**
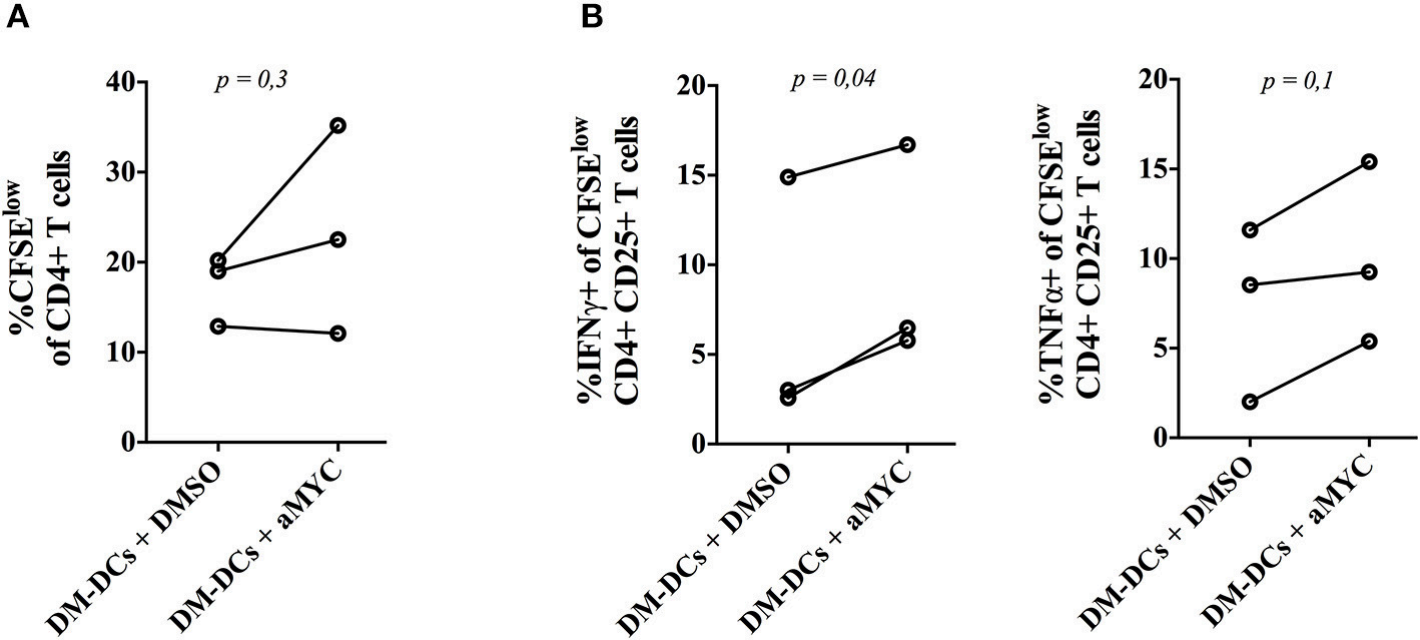
MYC blockade impairs Dex-modulated and MPLA-activated dendritic cells (DM-DCs) regulatory function. Functional regulatory capacity of DM-DCs was assessed by co-culturing DCs, differentiated with or without the MYC inhibitor 10058-F4, with allogeneic CD4+ T cells for 6 days. After that, T cell proliferation and cytokine expression was determined by flow cytometry. **(A)** Percentage of CFSE^low^ CD4+ T cells. **(B)** Percentage of IFNγ and TNFα producing T cells inside the proliferative CFSE^low^ CD4+CD25+ population. *n* = 3. DM-DCs, Dex-modulated MPLA-activated DCs+; M-DCs, MPLA-matured DCs.

Given that genes found to be modulated by Myc are proposed to contribute to the tolerogenic profile of DM-DCs, we set out to determine whether Myc inhibition could also affect the functional regulatory features of these cells. To evaluate this, we co-cultured DM-DCs that were differentiated in the presence of the Myc inhibitor 10058-F4 with allogeneic CD4+ T cells for 6 days and analyzed their ability to affect T cell activation and proliferation. DM-DCs normally display a reduced capacity to promote effector T cell proliferation and pro-inflammatory cytokine secretion (10, 11). Blockade of Myc during DM-DCs differentiation affects DM-DCs tolerogenic function, leading to an increase in CD4+ T cell proliferation (Figure 4A and Supplementary Figure S4), in addition to higher levels of IFNγ and TNFα expression in the proliferative CD25-activated CD4+ T cell population (Figure 4B and Supplementary Figure S4). These results support a role for Myc as an important modulator of DM-DCs tolerogenic features.

### Metabolic Pathways Enriched in DM-DCs

Myc is known to play an important role in many cellular processes, and more importantly, in metabolism. In the immune system, Myc acts as a key player coordinating metabolic reprogramming in different cell types (18, 19), and in DCs is a major regulator of energetic processes (19). Moreover, it could also be that a high expression of Myc in DCs is related with activation of metabolic processes.

**Figure 5:**
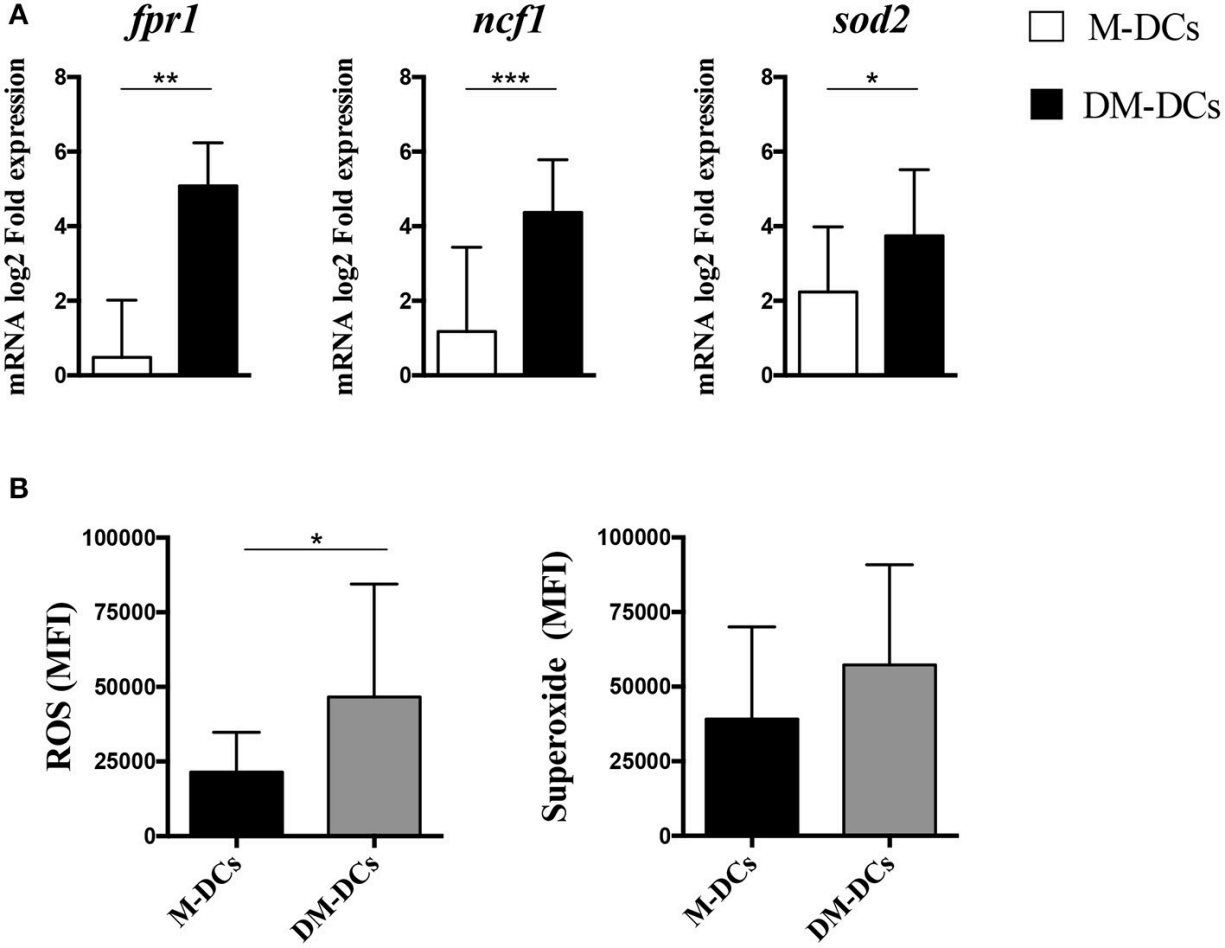
Intracellular concentration levels of reactive oxygen species and superoxide anions is higher in Dex-modulated and MPLA-activated dendritic cells (DM-DCs) than in other DC subtypes. Enrichment of ROS production in DM-DCs compared to DCs and M-DCs was confirmed through gene expression analysis of associated molecules and determination of intracellular levels of free radicals. **(A)** Determination of mRNA levels of genes related to ROS and superoxide production and upregulated on DM-DCs was determined by real time PCR. Data shows fold expression values of DM-DCs and M-DCs relative to untreated DCs expression (*n* = 10; *p = 0.05; **p = 0.01; **p = 0.001). **(B)** Total ROS and superoxide production in cells was assessed with two fluorescent cell-permeable reagents and flow cytometry analysis. Shown are MFI values for each probe reacting with ROS and superoxide (*n* = 3; *p = 0.05, **p = 0.01). DCs, untreated/immature DCs; DM-DCs, Dex-modulated MPLA-activated DCs+; M-DCs, MPLA-matured DCs; M-DCs, MPLA-matured DCs.

We have previously shown that several differentially expressed genes on DM-DCs are associated with functions guarding metabolic responses, mainly fatty acid metabolism, ROS production and ion homeostasis (12), all contributing in cellular balance. Several genes related to redox homeostasis and ROS production were amongst the most upregulated genes in DM-DCs [(12) and Supplementary Tables S3, S4], and as shown in Figure 5A, analysis of mRNA expression of different DC subsets using real time PCR confirms microarray analysis results, with DM-DCs exhibiting higher mRNA levels of these genes compared to the mature M-DCs control. To further confirm that this upregulation of redox-related genes has a functional outcome, we set to determine ROS intracellular levels on DCs, as a measure of evaluating cellular metabolic responses. As shown in Figure 5B, ROS levels are higher in DM-DCs than in M-DCs. Additionally, superoxide levels were also found to be higher in DM-DCs than M-DCs (Figure 5B), although the latter was not statistically significant; supporting an important role of redox metabolism in these cells.

### Zinc Influx Is Highly Regulated on DM-DCs

**Figure 6:**
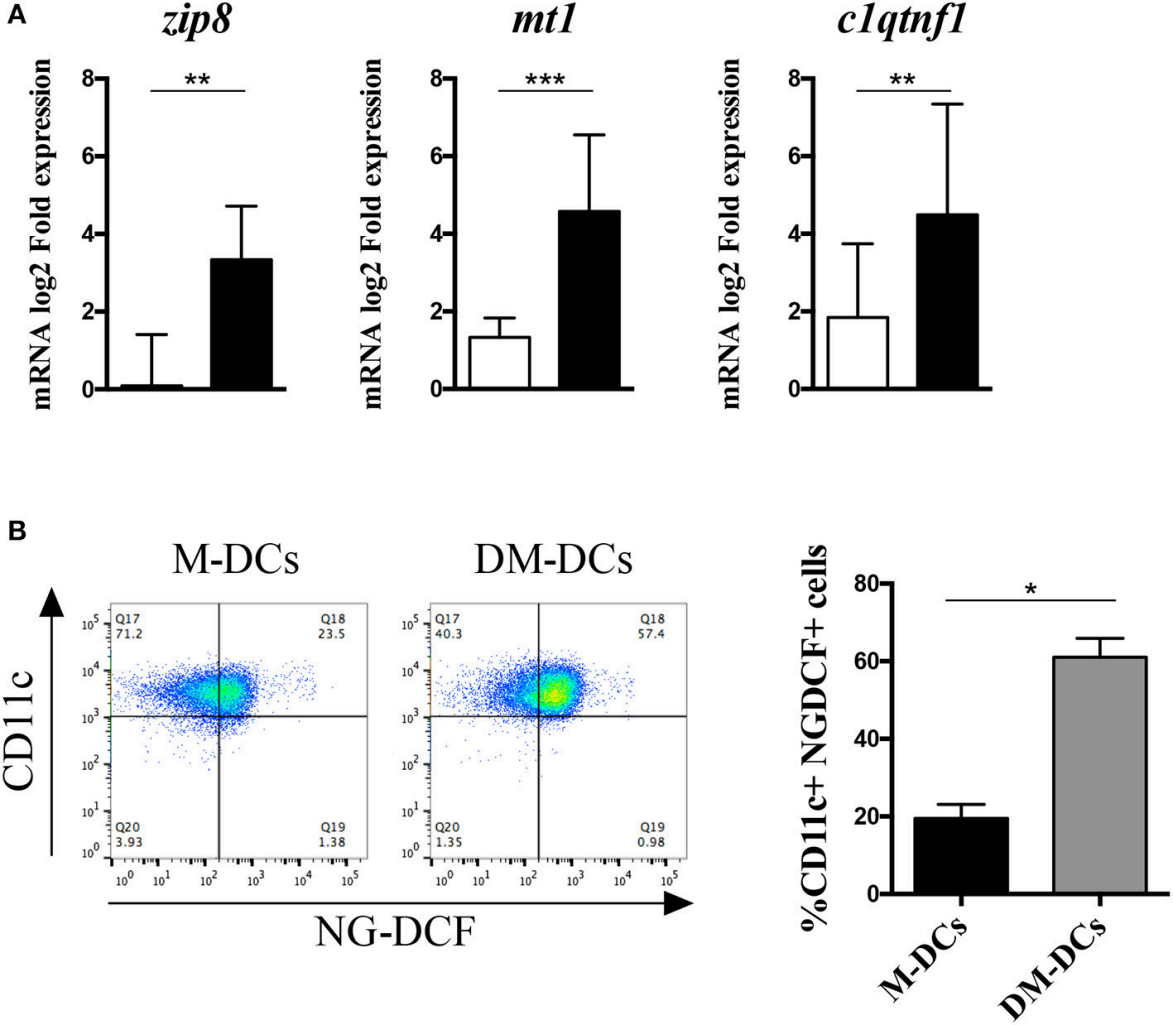
Dex-modulated and MPLA-activated dendritic cells (DM-DCs) show high expression of zinc transporters and zinc intracellular levels. Enrichment of zinc homeostasis in DM-DCs compared to DCs and M-DCs was evaluated. **(A)** Gene expression of zinc binding molecules upregulated in DM-DCs microarray analysis was determined by real time PCR. Data shows fold expression values of DM-DCs and M-DCs relative to untreated DCs expression (*n* = 10; *p = 0.05; **p = 0.01; **p = 0.001). **(B)** Determination of zinc intracellular levels in DCs was assessed with the zinc fluorescent indicator Newport Green DCF (NG-DCF) and analyzed by flow cytometry. Shown are percentages of CD11c+ cells positive for NG-DCF intracellular staining (*n* = 4; *p = 0.05, **p = 0.01). M-DCs, MPLA-matured DCs.

Besides being a metabolic product and therefore act as indicator of the cell's metabolic state, reactive oxygen species can act as second messengers in different signaling pathways controlling cellular proliferation and differentiation (20). One way by which ROS can drive changes in the cell is by increasing intracellular zinc levels through the modulation of the proteins involved in its cellular availability (21). In addition to ROS production, enrichment analysis of biological functions revealed that ion homeostasis, and cellular response to zinc ion in particular, were also highly represented on DM-DCs transcriptome (12), with several heavy metal scavengers and zinc binding proteins upregulated within these cells. Moreover, several genes involved in the regulation of this biological process, were not just upregulated, but remained amongst the most upregulated genes in DM-DCs transcriptome [Supplementary Tables S3, S4 and (12)]. Real time PCR analysis corroborated a higher expression of these genes in DM-DCs related to M-DCs and untreated DCs (Figure 6A), in most cases doubling mRNA levels, denoting an important role of these molecules in DM-DCs phenotype. Since all these genes coordinate zinc availability in the cell, we analyzed zinc intracellular levels in DCs using a cell-permeant fluorescent zinc probe (Supplementary Figure S5), and found that compared to M-DCs and untreated DCs, DM-DCs exhibited higher concentrations of the ion within its cytosol (Figure 6B).

## Discussion

The molecular setup driving the immune regulatory functions of tolerogenic DCs is still poorly understood. Even more, the transcriptional regulation of the genes involved in tolerance induction and regulatory responses of tolDCs remains to be fully comprehended. However, technological advances in the last years within the “omics” field as well as the development of multi-parametric flow cytometry analyses bring forward a deeper insight into the molecular characterization of DC biology (22–24). Using these techniques, we have previously described a set of tolerance-related markers found to be upregulated in Dex-modulated tolDCs, alternatively activated with MPLA (DM-DCs) at a transcriptional and at a protein level (12). Here, following the same approach, we identified a set of transcription factors differentially expressed in DM-DCs and potentially involved in the upstream modulation of tolerance markers upregulated in DM-DCs. In particular, we have found that the proto-oncogen MYC, is highly expressed in these cells and modulates the expression of the regulatory markers IDO1, JAG1 and MERTK, as well as other transcription factors STAT3 and PLZF, thus suggesting an important role of these molecule in DM-DCs tolerogenic features. Although the role of MYC in DC development and differentiation is well known (19, 25), a role for MYC in the establishment of immune regulatory responses had not been described before, except for the group of Casey et al. (26) whom showed that MYC promotes tumor growth and development through the modulation of the regulatory molecules CD46 and PD-L1 expression on tumor cells as a means to evade anti-tumor responses. PD-L1 is not expressed in our tolDCs (10), so we could not confirm its association with this regulatory marker, but we did find downregulation of other regulatory molecules, such as IDO1, JAG1 and MERTK when MYC was blocked. To our knowledge, this is the first time that a direct association between MYC and the immune regulatory phenotype of DCs has been established. Besides its effect on the expression of the three tolerance markers mentioned above, MYC blockade also led to downregulation of STAT3 and PLZF, transcription factors associated with immune regulation as well as inhibition of DC maturation (14), pointing out that MYC does not only directly controls the expression of tolerance genes on DM-DCs, but it could also indirectly modulate other regulatory genes and features through the modulation of these transcription factors. Even more, MYC inhibition also led to an impairment of DM-DCs functional features, further supporting its role as a modulator of DM-DCs tolerogenic profile.

In addition to upstream regulators analysis, IPA Functional Enrichment Analysis allowed us to discover that metabolic processes, mainly redox-related metabolic functions were highly represented on our cells, in particular ROS production and zinc homesotasis (12). Changes in cellular metabolism are key for certain aspects of DCs, and tolDCs have been described to display a different metabolic profile than other DC subtypes (27, 28). While inflammatory DC exhibit an anabolic type of metabolism which favors glycolysis, tolDCs show a highly energetic and catabolic profile, which in turn favors oxidative phosphorylation and fatty acid oxidation. Furthermore, it has been described that IL-10, a major hallmark of tolDCs, highly produced by our DM-DCs, can also favor this process through inhibition of the metabolic changes induced by TLR activation in DC (27, 29). In accordance to our previous IPA analysis findings (12), we not only confirmed a higher expression of genes involved in ROS and zinc homeostasis, but also found that these cells exhibited higher concentrations of both ROS and zinc in the cytosol, when compared to their mature (M-DCs) and immature counterparts (DCs).

ROS are known to participate in different physiological processes, and even though usually the presence of ROS and other free radicals is considered to promote pro-inflammatory responses, there are instances in which ROS has been shown to promote immune regulatory responses such as to exert a suppressive effect on immune cells (30). While effector T cells are sensitive to oxidative conditions, which if sustained leads to impaired proliferation and death of these cells, Treg can retain their suppressive features under the same circumstances. In fact, this is one of the strategies used by Treg as well as macrophages and other cells to suppress effector T cell responses and promote immune regulation (31–33). Even more, macrophages have been shown to induce Treg differentiation and proliferation in a ROS-dependent fashion (33), and both, macrophages and moDCs treated with Dex, an immunomodulatory drug used for immune modulation of inflammatory responses, also used by our group and others to generate tolDCs, are found to increase their ROS production, suggesting an association between the induction of a regulatory profile and ROS concentrations (34).

Zinc is known to modulate immune responses through its availability, while zinc deficiency can lead to increased inflammation and inflammatory diseases, zinc supplementation in DCs has been shown to interfere with their maturation, blocking surface expression of MHC II and co-stimulators, and promoting the expression of tolerance markers (35, 36). Yamasaki et al. (37) and Murakami and Hirano (38) propose zinc to act as an intracellular secondary messenger, which can affect other signaling molecules to modulate the final output triggered by extracellular stimuli. This effect depends on zinc availability, which in turn depends on two factors which differ in time, an early component such as a zinc wave that is induced by an extracellular stimuli, and a second component which depends on the transcriptional regulation of the expression of zinc transporters (38). Zinc availability in the cytosol is partly modulated by metallothioneins (MTs) in addition to other proteins such as zinc transporters, zinc-binding proteins and sensors (38). The treatment of DCs with dexamethasone and MPLA lead to the upregulation of several of the genes involved in zinc homeostasis, and gene expression analysis allowed us to confirm a higher expression of some of these molecules in DM-DCs compared to M-DCs and untreated DCs. Furthermore, to confirm a functional relation between zinc-binding proteins and zinc availability, we determined zinc intracellular levels on different DCs subtypes, finding a higher zinc influx on DM-DCs than in the mature (M-DCs) or immature control (DCs).

Several forms of MTs, cysteine-rich metal binding proteins involved in the homeostasis of zinc and other heavy metals at a cytoplasmic level, displayed the highest fold change values and were highly represented in DM-DCs transcriptomic profile [Tables S3, S4 and (12)]. Both, MTs expression and function, is modulated by intracellular ROS. Oxidation of MTs leads to release of zinc ions intro the cytosol, increasing the influx of this cation in the cell. Thus, MTs fulfill an important role as signal transducers, turning ROS signals into transient increases of zinc (21) which then can act as an intracellular secondary messenger as described by Yamasaki et al. (37).

Thus, our work shows that the tolerogenic features of DM-DCs are partially modulated by the expression of MYC, as well as ROS production and zinc influx. This proposed regulatory role of MYC on DM-DCs could not only be occurring on our DCs. A review made by our group regarding transcriptional and proteomic research developed in human tolDCs, revealed that this transcription factor was expressed in almost all tolDCs studied (23), further supporting our findings for an important role of this transcription factor in tolDCs.

MYC is a key regulator of many biological processes within the cell, mainly metabolic programs, and it has been described that its expression increases intracellular ROS levels (39–43). In addition to their potential role in immune regulation and apart from acting as an indicator of the cell's metabolic state, ROS can act as second messengers in different signaling pathways controlling cellular proliferation and differentiation (20). One way by which ROS can drive changes in the cell is by increasing intracellular zinc levels through the modulation of proteins involved in its cellular availability (21). In DCs, besides its importance in development and differentiation, MYC has an important role in DC:T cell priming and DC metabolism (18, 43, 44), so we propose that there is a close association between all findings of this work, where MYC expression not only leads to transcriptional regulation of tolerance markers, but also to increase in ROS levels, which in turn is translated into a zinc signal. However, further studies will be required to confirm this.

## Ethics Statement

All subjects signed an informed written consent and all procedures were approved by the Ethics Committees for Research in Human Beings from the Faculty of Medicine and from the Clinical Hospital of the University of Chile.

## Author Contributions

PG-G, KS, JA, RV, RT, and DC participated in the conception and design of the study. PG-G, KS, JA, RV, AS-G, DC, OA, and MM worked on analysis and interpretation of data. PG-G and JA participated in manuscript preparation and redaction. PG-G, KS, AM, HN, AS-G, and JM performed most experiments and data acquisition. LS, ON, and JM participated in the recruitment of subjects for this study. BP collaborated in data acquisition and analysis.

### Conflict of Interest Statement

The authors declare that the research was conducted in the absence of any commercial or financial relationships that could be construed as a potential conflict of interest.
