# Supplementary figures and images for: Regulation of Tolerogenic Features on Dexamethasone-Modulated MPLA-Activated Dendritic Cells by MYC
Source: Front Immunol. 2019 May 28;10:1171. doi: 10.3389/fimmu.2019.01171 (PMC6547838; doi:10.3389/fimmu.2019.01171)

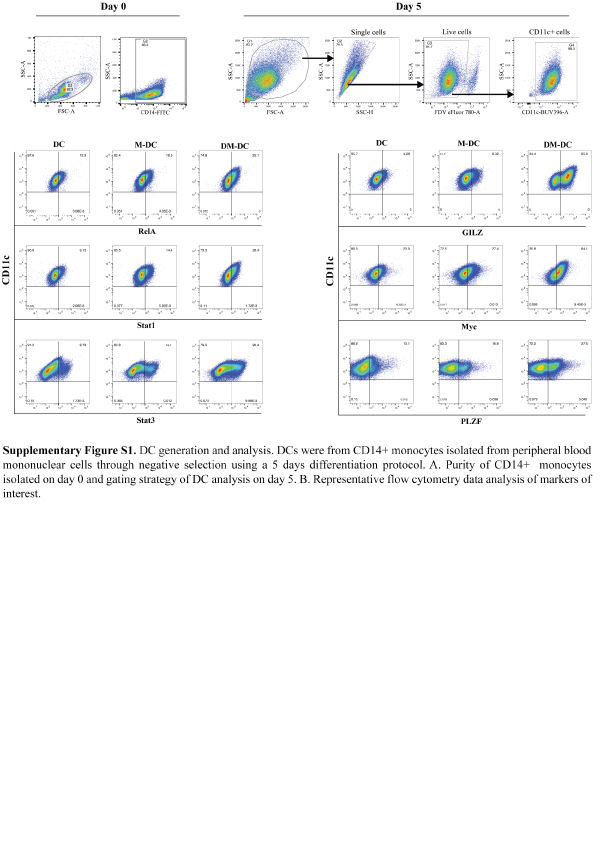

Supplement: Supplementary file 5 [file Image_1.jpg]

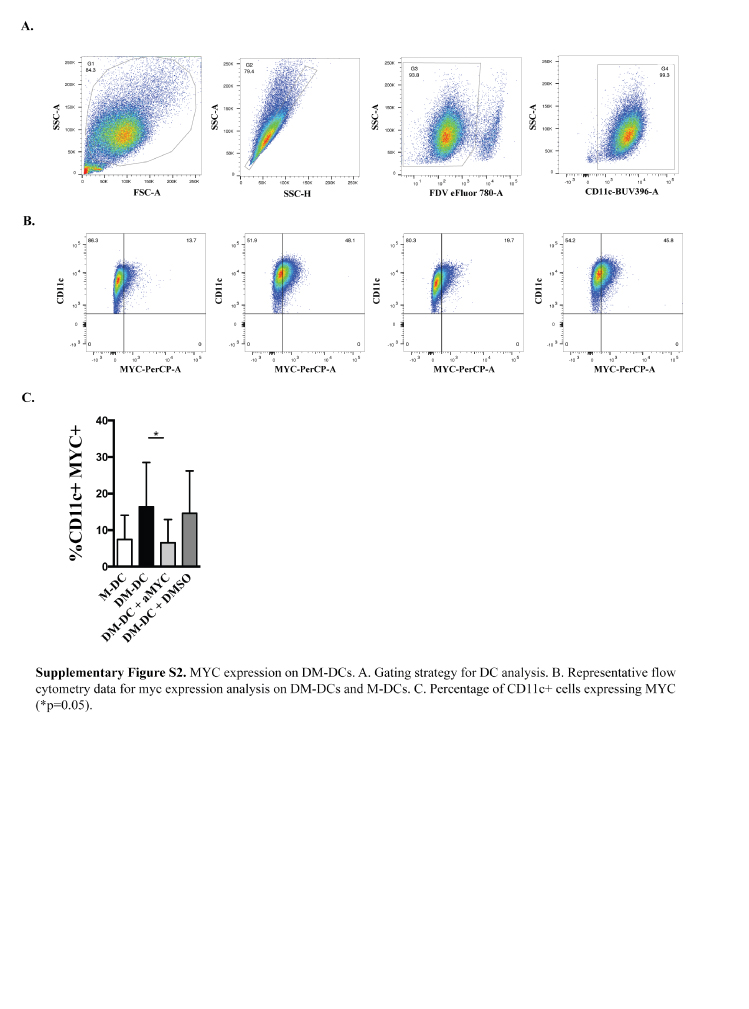

Supplement: Supplementary file 6 [file Image_2.jpg]

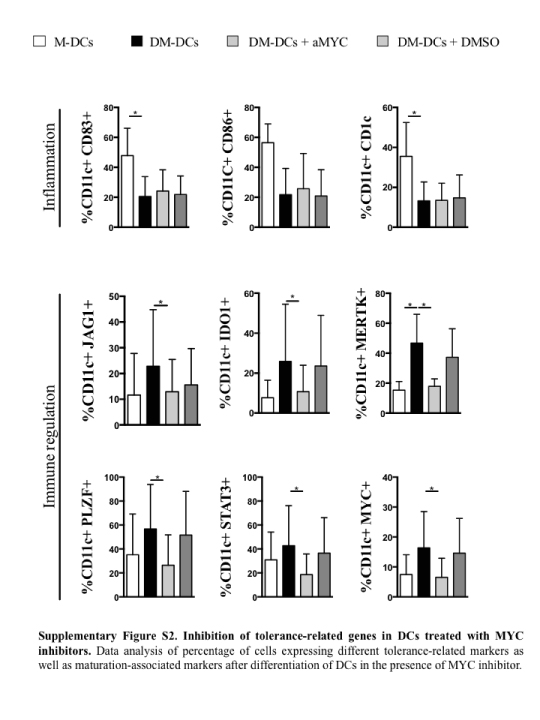

Supplement: Supplementary file 7 [file Image_3.jpg]

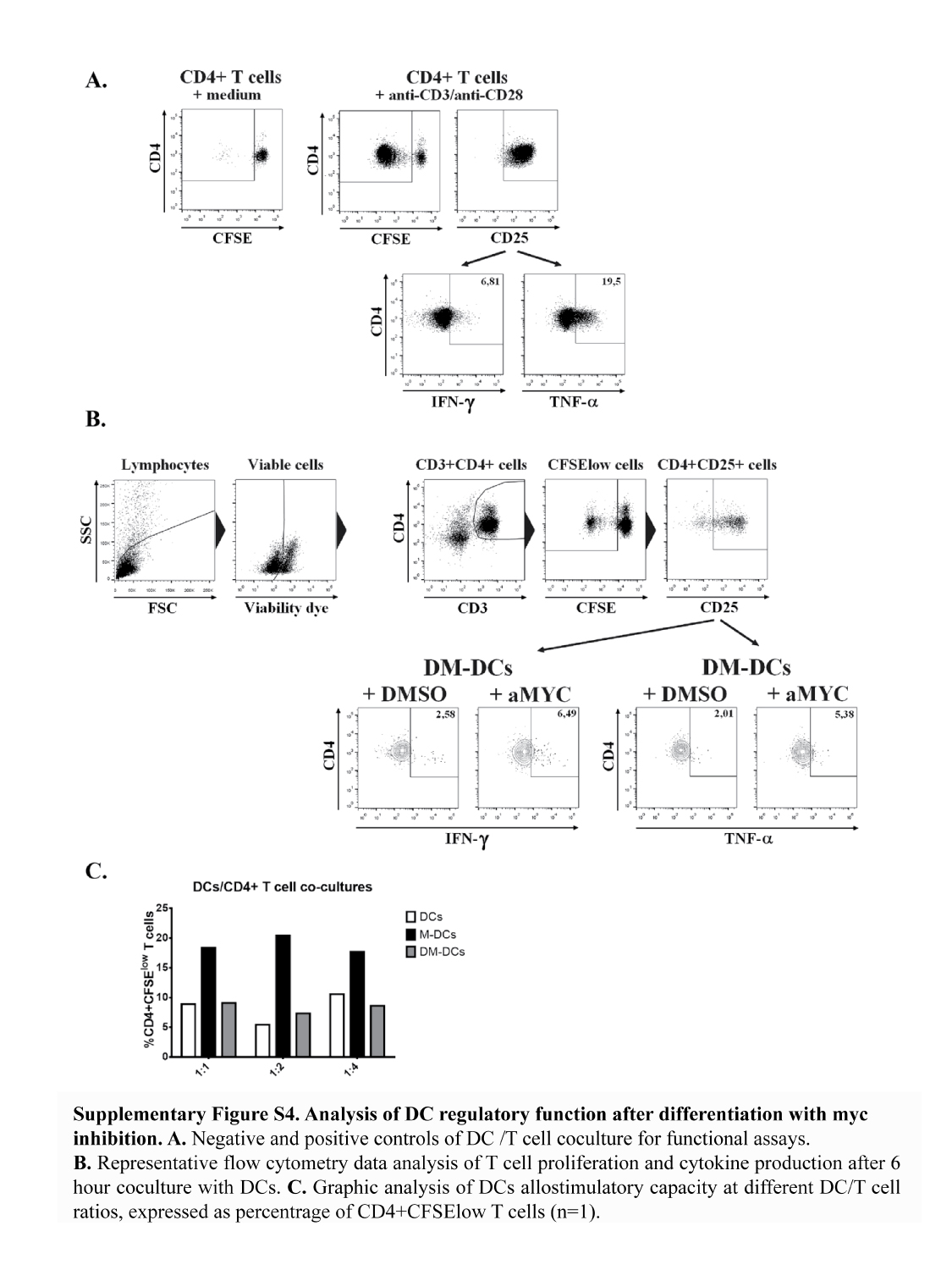

Supplement: Supplementary file 8 [file Image_4.jpg]

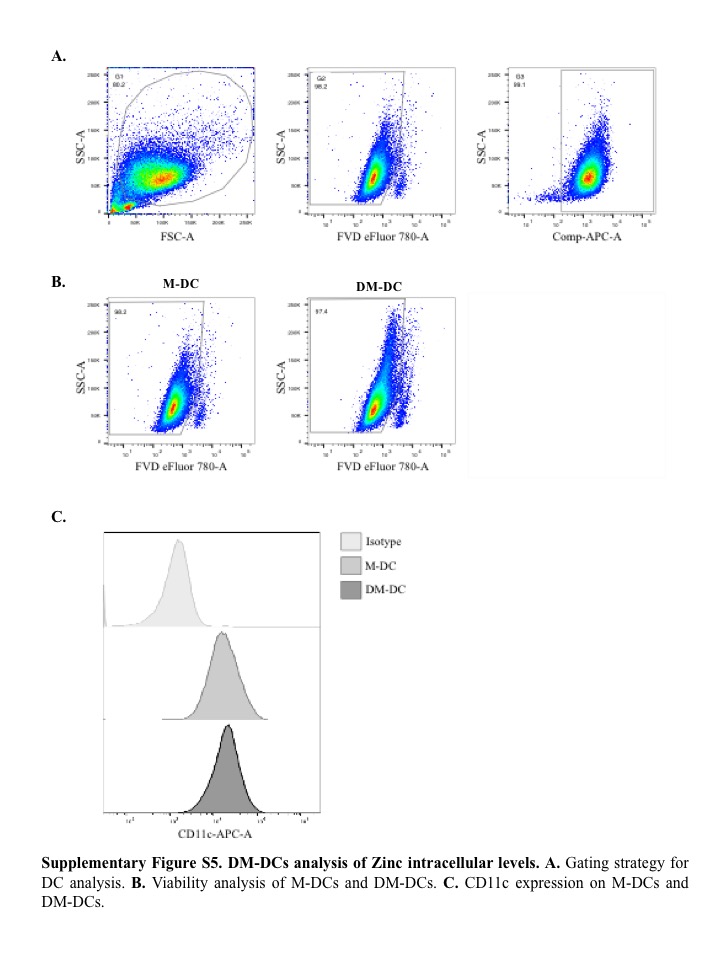

Supplement: Supplementary file 9 [file Image_5.jpg]
